# The effect of HLA matching and donor relatedness on the risk of autoimmune haemolytic anaemia in haematopoietic stem cell transplant recipients: A systematic review and meta‐analysis

**DOI:** 10.1002/jha2.509

**Published:** 2022-07-15

**Authors:** Cassandra Kennedy, Denise E. Jackson

**Affiliations:** ^1^ Discipline of Laboratory Medicine School of Health and Biomedical Sciences STEM College RMIT University Bundoora Victoria Australia

**Keywords:** AIHA, donor relatedness, HLA‐matching, HSCT

## Abstract

Recent studies have identified autoimmune haemolytic anaemia (AIHA) as a haematopoietic stem cell transplant (HSCT) complication that represents a significant cause of morbidity and mortality for these patients. In order to understand this autoimmune phenomenon, emerging research has focused on the prognostic factors associated with the development of the disorder. These studies have identified numerous possible associations with often contrasting and conflicting results. A systematic review and meta‐analysis were performed in order to determine the effect of human leucocyte antigen (HLA) matching and donor relatedness on the risk of AIHA post‐HSCT. PubMed, SCOPUS and ProQuest were searched from 1 January 1995 to 1 August 2021 using a range of keywords. Meta‐analysis was performed using OpenMeta‐Analyst software using a random effects model and arcsine risk difference (ARD). Eight eligible articles were identified, and meta‐analysis showed an increased risk of AIHA in those who received HLA‐mismatched transplants (ARD −0.082; 95% confidence interval [CI] −0.157, −0.007; *p* = 0.031) and those who received donations from unrelated donor sources (ARD −0.097; 95% CI −0.144, −0.051; *p* < 0.001). Patients who receive HSCT from HLA‐matched and related donor sources have a reduced risk of developing AIHA. Healthcare practitioners should be mindful of the risk of AIHA, especially in those who receive HLA‐mismatched and unrelated donor‐sourced stem cells. While these findings provide further evidence for researchers investigating the pathogenesis of this HSCT complication, more studies are needed to fully understand the cause.

## 1 INTRODUCTION

Autoimmune haemolytic anaemia (AIHA) is a condition in which an autoimmune response is mounted against the patient's own red cell antigens, leading to haemolysis [1]. Recently, AIHA has emerged as a relatively common complication of haematopoietic stem cell transplant (HSCT), with an incidence of approximately 1%–6% depending on the associated population [1, 2, 3]. Previous studies have found that this complication is especially prevalent in paediatric populations, and there is limited evidence that it may be impacted by donor and recipient factors such as human leucocyte antigen (HLA) matching and donor source [4, 5, 6, 7, 8]. In addition, posttransplant AIHA is especially problematic, as it is often refractory to treatment and is associated with a high risk of mortality and morbidity for the patient [5]. Wang et al. [5] found that patients who present with AIHA posttransplant have double the risk of mortality as those without autoimmune complications. Therefore, AIHA in the posttransplant setting represents a significant source of mortality and morbidity for allogeneic HSCT recipients, and further understanding of the associated risk factors for the condition is imperative to understanding the pathophysiology and aetiology of this complication.

### 1.1 Autoimmune haemolytic anaemia: Aetiology, pathophysiology and diagnosis

Classically, AIHA can present as a primary condition or may be secondary to another condition, such as infection or malignancy. Furthermore, AIHA can be classified according to the class of autoantibodies that are produced in the patient and its thermal amplitude, including warm AIHA (wAIHA), cold AIHA (cold agglutinin disease [CAD]), mixed AIHA and atypical forms [2]. Typically, wAIHA, which includes immunoglobulin G (IgG) antibodies that react at 37°C, is the most common, with up to two‐third of patients presenting with this form [1]. In contrast, cold AIHA generally involves IgM antibodies that react at room temperature and typically account for one‐third of AIHA cases [1]. Approximately 5% of patients present with mixed‐type AIHA, which can present with both antibody classes.

The aetiology of AIHA is thought to be associated with a complex interaction between environmental factors and genetic predispositions [9]. Typically, autoimmunity is thought to be associated with a breakdown of tolerance due to the presence of autoreactive B and T cells [9]. Studies in animal models have suggested that reduced CD4^+^ and CD25^+^ Treg cells may be responsible for the escape of autoreactive lymphocytes; however, further studies have suggested that these cells are not required to prevent the development of AIHA [10, 11]. In further studies, the link between the balance of Th17 and Treg cells has been investigated as being the source of the autoimmunity seen in AIHA [12]. Th17 cells are typically thought to be associated with the development of autoimmunity and inflammation and are known to release the proinflammatory cytokine interleukin‐17 (IL‐17) [12]. Xu et al. [12] demonstrated that Th17 cells also play a critical role in the development of AIHA, with increased levels of the cells in those with the disease. Furthermore, a correlation was also observed between the levels of IL‐17 and the associated disease activity of patients with AIHA [12]. Importantly, some cytokine gene polymorphisms have also been found to be associated with the Th17/Treg imbalance seen in AIHA [9].

Once the immune dysregulation seen in AIHA has triggered the production of autoantibodies to red blood cell (RBC) antigens, immune‐mediated destruction of red cells is seen [13]. Typically, wAIHA results in the production of IgG antibodies that react at body temperature. While the antibodies may also weakly bind complement, the majority of associated haemolysis is due to extravascular haemolysis associated with the monocyte‐macrophage system phagocytosing RBCs through the Fc portion of the IgG attached to the RBC [13]. In contrast, IgM‐associated autoantibodies in AIHA are capable of activating intravascular haemolysis through the activation of the complement cascade. Furthermore, IgM can also trigger extravascular haemolysis through the phagocytosis of cells with bound complement by Kupffer cells in the liver, as seen in CAD [13]. Importantly, with AIHA, the thermal amplitude of autoantibodies also determines their clinical significance, with IgG antibodies typically being associated with reactions at body temperature (approximately 37°C) presenting with significant haemolysis and IgM typically being associated with reactions at room temperature (4°C–34°C) and therefore typically being associated with a more benign course [13].

Diagnosis of AIHA is typically by a direct antiglobulin test (DAT), which demonstrates the presence of autoantibodies, and/or complement, on the surface of patient red cells. In addition, indirect antiglobulin tests (IATs) are often performed on the antibodies eluted from DAT‐positive red cells (i.e., the eluate) to determine if there is any specificity of the autoantibody present in the serum [14]. Typically, wAIHA will present with DAT positivity for IgG, with or without complement, whereas CAD is usually positive for complement alone [14]. Mixed‐form AIHA can have both IgG and IgM present and therefore may have DAT positivity with IgG, complement or both. In addition, however, atypical forms of AIHA exist, which include DAT‐negative forms, IgA‐driven forms and warm IgM forms [14]. Further complicating matters, DATs can often be falsely positive due to various therapies, including intravenous immunoglobulins [14]. Along with the detection of the antibody, or complement, coating the red cell, tests for associated haemolysis are also undertaken to determine the degree of red cell destruction, including blood film examination, reticulocyte counts, lactate dehydrogenase (LDH), haptoglobin and bilirubin [9].

### 1.2 Autoimmune haemolytic anaemia as a HSCT complication

In the post‐allogeneic HSCT transplant setting, autoimmunity refers to donor lymphocytes that target donor‐derived tissue or antigens common between both the donor and recipient [6]. Autoimmune reactions after HSCT are a relatively common complication, with AIHA having the highest incidence of approximately 1%–6% [1, 2, 3]. Typically, these AIHA cases are wAIHA, frequently with a pan‐reactive antibody, and are often resistant to treatment [3, 6]. The pathophysiology of these cases of AIHA is not well understood; however, there have been suggestions that these may be cases of the donor immune system reacting again to mismatched self‐antigens; however, the evidence to support this has been mixed [6]. Chimerism studies performed by Wang et al. support the interpretation that these conditions are a ‘donor against donor’ immune complication [5].

In order to understand the pathophysiology of post‐HSCT AIHA, studies have investigated the effect of HLA mismatch and the relatedness of donor sources on the development of AIHA. Previous studies have identified haploidentical related donors, unrelated donors and HLA mismatch as significant risk factors for the development of AIHA posttransplant; however, other studies have not seen the same effect of these risk factors [3, 4, 5, 6, 7, 8]. Additionally, other risk factors have been identified, such as the presence of cytomegalovirus (CMV) infection, chronic graft versus host disease and paediatric age [4, 7]. Although the results have been mixed, a thorough understanding of the risk factors associated with the development of this autoimmune condition may inform future transplant protocols and help to elucidate the complex pathophysiology underlying these conditions. Therefore, the aim of this meta‐analysis and systematic review is to investigate the effects of HLA disparity and relatedness of donor source stem cells on the rates of AIHA in adult and paediatric patients post‐allogeneic HSCT through the examination of retrospective studies. We hypothesise that transplants with a greater HLA disparity and from an unrelated source will be associated with a greater risk of AIHA.

## 2 METHOD

### 2.1 Study design

In this systematic review, the protocols outlined in the Preferred Reporting Items for Systematic Reviews and Meta‐analysis (PRISMA) were adopted to guide the search and identification of articles investigating the effects of HLA matching and donor source of haematopoietic stem cells on the risk of AIHA in adult and paediatric HSCT recipients [15]. In addition, these articles were assessed for their quality through use of the Strengthening the Reporting of Observational Studies in Epidemiology (STROBE) checklist [16].

### 2.2 Search strategy

**FIGURE 1:**
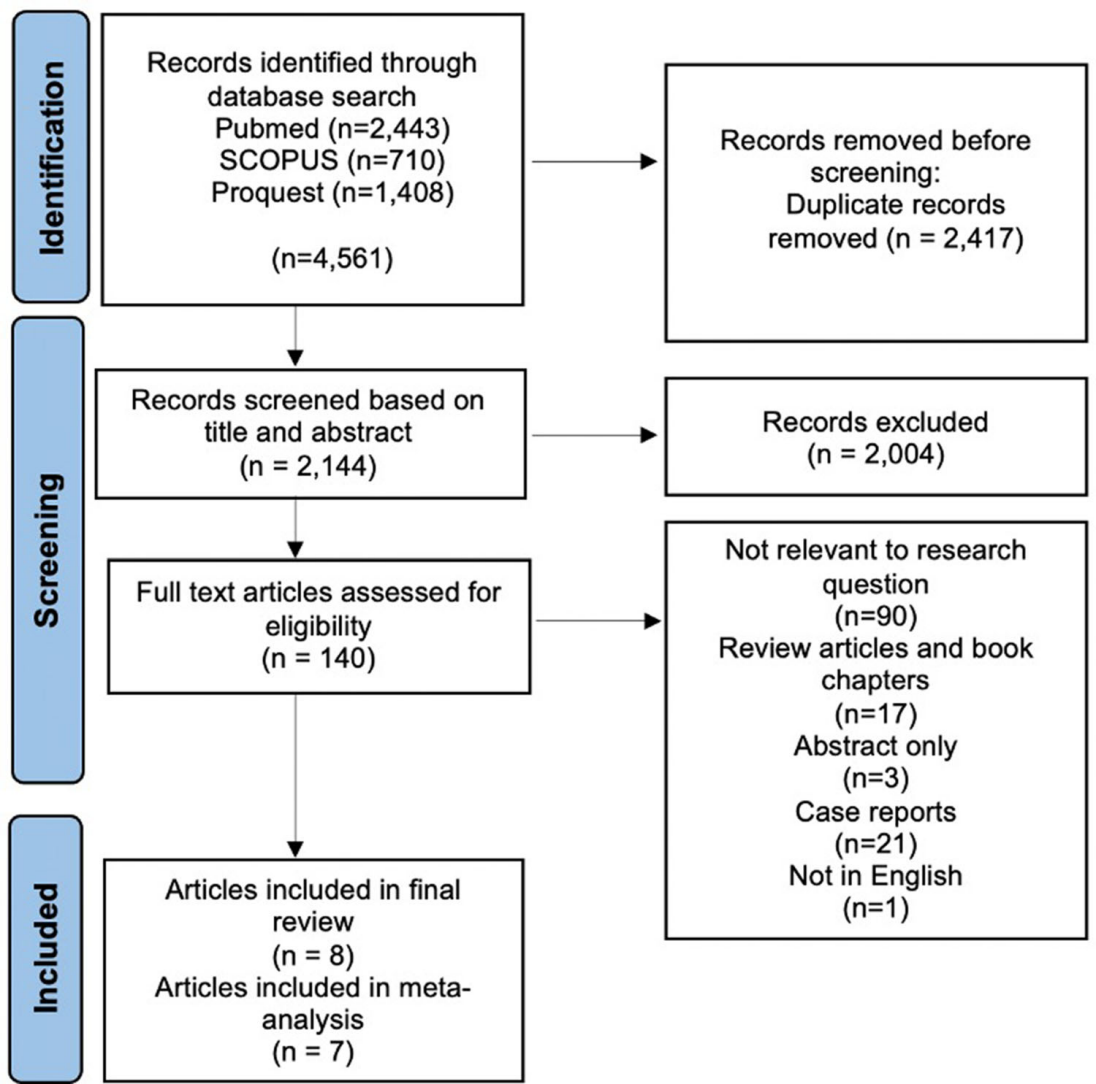
Preferred Reporting Items for Systematic Reviews and Meta‐analysis (PRISMA) flowchart of study identification. Studies were selected based on a thorough search of three databases, along with a manual search. Following the removal of duplicates, studies were screened based on title and abstract. Further studies were determined for eligibility through a series of criteria. Articles that were not excluded were included in the final systematic review

In order to identify the appropriate literature, PubMed, SCOPUS and ProQuest were systematically searched from 1 January 1995 to 1 August 2021, using a range of keywords and phrases. These phrases included various combinations of ‘AIHA’, ‘immune anaemia’, ‘autoantibody’ AND ‘stem cell transplant’, ‘allogeneic stem cell transplant’, ‘haematopoietic stem cell transplant’, ‘bone marrow transplant’, ‘related bone marrow transplant’, ‘unrelated donor bone marrow transplant’, ‘matched HLA transplant’ and ‘HLA mismatched transplant’. Additional papers were manually searched for in the reference lists of relevant literature; however, no further eligible studies were identified. The resultant search result numbers are included in the PRISMA flowchart in Figure 1.

### 2.3 Eligibility criteria

Articles identified through the previously listed search strategy were screened through their title and abstract using a set of predetermined inclusion and exclusion criteria to determine eligibility. Articles that examined the effect of HLA matching and related versus unrelated donor sources on the development of posttransplant AIHA in allogeneic stem cell transplant recipients were deemed eligible. Articles were excluded based on several criteria, including: (1) review, meta‐analysis, or meeting/conference proceedings, (2) language: not in English, (3) not examining AIHA rates specifically, (4) did not examine effect of HLA matching or relatedness of donor source, (5) not including a comparison group for either outcome, (6) examining treatment or survival rates for AIHA or stem cell transplants, and (7) abstract only papers.

### 2.4 Assessment of methodological quality

Each study that was identified as eligible was assessed for methodological quality through the use of the published standards outlined by the STROBE guidelines, as shown in Table 3 [16].

### 2.5 Data extraction

The relevant data from each eligible study were tabulated with the listed primary author, year of publication, study design and period and the incidence of AIHA found in the study, as shown in Table 1. In addition, the risk factors that were identified as being significantly associated with an increased risk of AIHA were listed, if applicable. An additional table was constructed that showed the number of patients who did and did not develop AIHA in the unrelated and related donor stem cell groups, as well as the HLA‐matched and HLA‐mismatched groups.

**TABLE 1:** Characteristics of eligible studies included in the analysis of autoimmune haemolytic anaemia (AIHA) risk in recipients of haematopoietic stem cell transplant (HSCT) from related versus unrelated sources and HLA‐matched versus unmatched sources

Study	Design	Study period	Country	Population	Study size	Incidence of AIHA	Risk factors identified as significant
Lv et al. 2019 [3]	Retrospective	2011–2016	China	Adult	1377	2.2%	Haploidentical related donors, cGVHD
Sanz et al. 2007 [6]	Retrospective	1996–2004	Spain	Adult	272	4.4%	Unrelated donors and cGVHD
Wang et al. 2015 [5]	Retrospective	2005–2011	UK	Adult	533	3.6%	Unrelated donors
Gonzalez et al. 2018 [4]	Retrospective	2000–2015	Spain	Adult and paediatric	4099	1.5%	Paediatric age, use of cord blood, HLA‐mismatched donor
Yang et al. 2014 [7]	Retrospective	2010–2012	China	Adult and paediatric	296	4.1%	CMV, GVHD, HLA mismatch
O'Brien et al. 2004 [8]	Retrospective	1995–2011	USA	Paediatric	439	6%	Metabolic disease, unrelated donors
Ahmed et al. 2015 [19]	Retrospective	11‐year period^a^	USA	Paediatric	500	2.4%	No single risk factor^b^
Chang et al. 2016 [18]	Retrospective	1998–2015	Taiwan	Paediatric	265	6%	None

Abbreviations: cGVHD, chronic graft versus host disease; CMV, cytomegalovirus; HLA, human leucocyte antigen.

^a^ Years not specified.

^b^ Transplants that were both HLA matched and from related donors were found to significantly decrease the risk of AIHA compared to other donor sources.

### 2.6 Data synthesis and statistical analysis

The use of OpenMeta‐Analyst software (version 12.11.14), obtained from the Brown University website, was employed to synthesise the data and conduct the meta‐analysis [17]. The data displayed in Table 2 were entered according to the associated exposure. A two‐armed proportion analysis, using a difference in arcsine transformed proportion, was employed using a binary random effects model with a maximum‐likelihood random effects method. A forest plot was constructed, along with the calculation of the 95% confidence intervals, *p*‐values and heterogeneity using OpenMeta‐Analyst software. A *p*‐value of less than 0.05 was defined as ‘statistically significant’.

**TABLE 2:** Data of eligible studies included in the meta‐analysis reporting the proportion of autoimmune haemolytic anaemia (AIHA) cases for recipients of HLA‐matched and unmatched haematopoietic stem cell transplant (HSCT) and related and unrelated donor HSCT

Study	Donor source	Patients with AIHA	Patients without AIHA	HLA matching	Patients with AIHA	Patients without AIHA
Lv et al. 2019 [3]	Related	6	760	Matched	11	1045
	Unrelated	5	323	Mismatched	15	306
Sanz et al. 2007 [6]	Related	5	189	Matched	6	190
	Unrelated	7	71	Mismatched	6	70
Wang et al. 2015 [5]	Related	2	179	Matched	15	402
	Unrelated	17	335	Mismatched	4	112
Yang et al. 2014 [7]	Related	1	148	Matched	4	264
	Unrelated	3	112	Mismatched	7	20
O'Brien et al. 2004 [8]	Related	0	136	Matched	10	129
	Unrelated	19	284	Mismatched	9	155
Ahmed et al. 2015 [19]	Related	5	257	Matched	5	336
	Unrelated	7	231	Mismatched	7	140
Chang et al. 2016 [18]	Related	1	72	Matched	6	106
	Unrelated	14	178	Mismatched	9	144

## 3 RESULTS

### 3.1 Study selection

Using the predetermined search strategy, 4561 citations were retrieved from electronic search databases, and the manual search did not find any additional relevant references. Duplicates were then removed, which resulted in a remaining total of 2144 studies. These studies were screened based on the title and abstract, and an additional 2004 were considered ‘not relevant’ to the research question. The remaining 140 studies were thoroughly examined through their full‐text articles, and using the exclusion criteria, 132 were excluded from the final review. The number of studies excluded based on each criterion is listed in Figure 1. Of the remaining eight articles, seven were used in the meta‐analysis, and one was not included in this quantitative analysis due to a failure to include sufficient data regarding total exposure numbers of HLA‐matched and related/unrelated transplant in the study cohort.

### 3.2 Study characteristics

A total of eight eligible studies were identified that examined the link between AIHA risk and HLA matching and the relatedness of donors in HSCT [2, 3, 4, 5, 6, 7, 8, 18, 19]. As shown in Table 1, these studies were all retrospective in nature and spanned five countries. Of the studies included, three were exclusively adult populations, whereas two included adult and paediatric patients together [3, 4, 5, 6, 7]. In addition, two studies included only paediatric patients [8, 19]. In the eligible studies, patients were mainly seeking transplant for the treatment of haematological disorders and malignancies [3, 5, 6]; however, studies that included paediatric patients also included patients being treated for immunodeficiencies, metabolic diseases and thalassaemia [4, 7, 8, 18, 19].

AIHA was defined slightly differently in the various eligible studies. Of the included studies, three defined AIHA as (a) a positive DAT, (b) clinical and laboratory evidence of haemolysis, (c) a pan‐reactive IAT with the eluate, and (d) other possible immune‐mediated causes of haemolysis excluded [3, 6, 19]. In contrast, an additional three studies included a similar definition; however, they did not require a positive eluate to diagnose AIHA [4, 5, 8]. The remaining two studies used unique definitions of AIHA, including one defining an AIHA case as a patient having symptoms of anaemia and haemolysis along with a positive DAT [18] and one requiring a decreased haemoglobin, reticulocytosis and elevated LDH and bilirubin along with a positive DAT [7]. Six of the studies did not include patients with a previous history of AIHA or positive DAT [3, 5, 6, 8, 19], whereas the others did not specify if a history of positive DAT resulted in ineligibility in the study.

### 3.3 Methodological quality assessment

The articles included in this systematic review were analysed for their methodological quality using the STROBE criteria, with the most relevant criteria summarised and presented in Table 3. Of the analysed studies, most were of high quality and fulfilled most of the criteria. While most were identified as being of a high quality, one study was found to be of a lower quality, as it had not sufficiently outlined the total numbers of each type of transplant undertaken in the study [4]. Therefore, this study failed to give characteristics of all study participants and failed to define all exposure. In addition, it also failed to discuss the limitations of the study [4]. Apart from this study, the remaining studies greatly satisfied the vital criteria presented in Table 3. However, it should be noted that three studies failed to address potential bias by not excluding patients with a history of AIHA or positive DAT prior to HSCT [4, 5, 6]. In addition, four identified studies failed to explicitly state the source of their funding [4, 5, 6, 8]. Regardless, the included studies were found to be mostly of high methodological quality, which suggests that the risk of bias introduced through inappropriate study selection is low.

**TABLE 3:** Evaluation of methodological quality of included studies according to the Strengthening the Reporting of Observational Studies in Epidemiology (STROBE) checklist

	Lv et al. 2019 [3]	Sanz et al. 2007 [6]	Wang et al. 2015 [5]	O'Brien et al. 2004 [8]	Gonzalez et al. 2018 [4]	Ahmed et al. 2015 [19]	Yang et al. 2014 [7]	Chang et al. 2016 [18]
Title and abstract								
Clear title and abstract with information on study design	Y	Y	Y	Y	Y	Y	Y	Y
Introduction								
Explains scientific background	Y	Y	Y	Y	Y	Y	Y	Y
Methods								
Describes setting, relevant dates and exposure	Y	Y	Y	Y	Y	N^a^	Y	Y
Defines all outcomes and exposures	Y	Y	Y	Y	N^b^	Y	Y	Y
Describes any efforts to address potential bias	Y^c^	Y^c^	Y^c^	Y^d^	N	Y^c^	N	N
Describes statistical methods	Y	Y	Y	Y	Y	Y	Y	Y
Results								
Give characteristics of study participants	Y	Y	Y	Y	N^e^	Y	Y	Y
Discussion								
Summarise key results and discusses limitations	Y	Y	Y	Y	N^f^	Y	N^f^	Y
Gives source of funding	Y	N	N	N	N	Y	Y	Y

*Note*: Y = criteria fulfilled; N = criteria not fulfilled.

^a^ Does not provide relevant dates, only specifies 11 years.

^b^ Does not give total overall exposure numbers.

^c^ Patients with a history of autoimmune haemolytic anaemia (AIHA) were excluded.

^d^ Did not include patients with a history of positive direct antiglobulin test (DAT) or AIHA.

^e^ Only listed characteristics for AIHA group.

^f^ No limitations discussed.

### 3.4 Meta‐analysis of HLA matching and risk of AIHA

**FIGURE 2:**
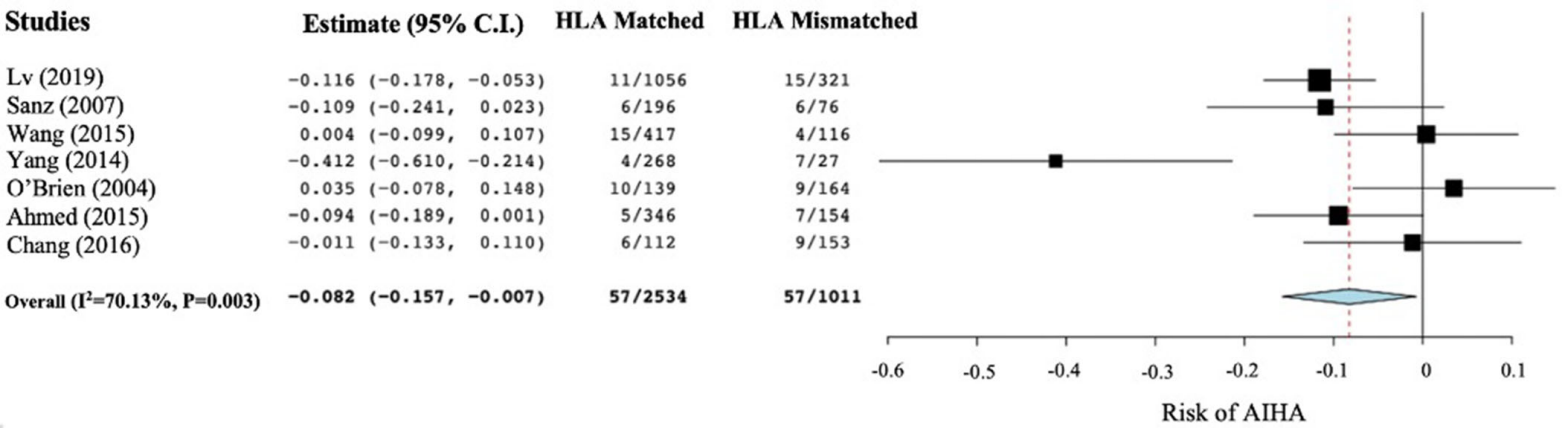
Forest plot of meta‐analysis on the effect of HLA matching on the risk of autoimmune haemolytic anaemia (AIHA) in haematopoietic stem cell transplant (HSCT) recipients. Data on AIHA incidence were extracted as the proportion of AIHA cases in those who were HLA matched or mismatched over the total number of HLA‐matched or mismatched transplants performed in each study. These were then used to perform a difference in arcsine proportion analysis, which was then expressed as an arcsine risk difference (ARD), and pooled effect size was calculated as ARDs with 95% confidence intervals (CI). The statistical significance was calculated as *p*‐values. The heterogeneity of the included studies was calculated as an *I* ^2^ value with an associated *p*‐value. Weighting of each study was determined through the relative sample sizes

A meta‐analysis and forest plot of the effect of HLA matching on the incidence of AIHA was conducted, as shown in Figure 2. The two‐arm proportion analysis found that there was an overall decreased risk of AIHA in those who received HLA‐matched HSCT when compared with those who received an HLA‐mismatched transplant (arcsine risk difference [ARD] −0.082; 95% confidence interval [CI] −0.157, −0.007; *p* = 0.031). However, the included studies were found to have substantial heterogeneity (*I* ^2 ^= 70.13%; *p* = 0.003).

### 3.5 Meta‐analysis of the relatedness of donors and the risk of AIHA

**FIGURE 3:**
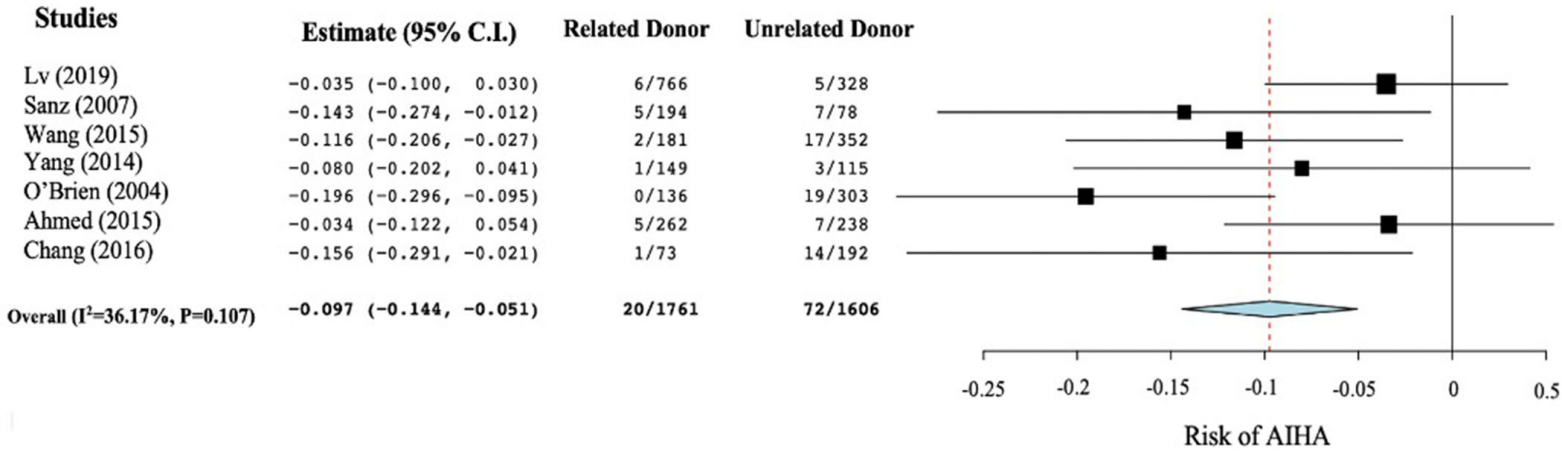
Forest plot of meta‐analysis on the effect of donor relatedness on the risk of autoimmune haemolytic anaemia (AIHA) in haematopoietic stem cell transplant (HSCT) recipients. Data on AIHA incidence were extracted as the proportion of AIHA cases in those who were HLA matched or mismatched over the total number of HLA‐matched or mismatched transplants performed in each study. These were then used to perform a difference in arcsine proportion analysis, which was then expressed as an arcsine risk difference (ARD) with pooled effect size calculated as ARDs with 95% confidence intervals (CI). The statistical significance was calculated as *p*‐values. The heterogeneity of the included studies was calculated as an *I* ^2^ value with an associated *p*‐value. Weighting of each study was determined through the relative sample sizes

The effect of donor relatedness on the incidence of AIHA was examined through a meta‐analysis and forest plot, as shown in Figure 3. A two‐arm proportion analysis was conducted on the data, and there was found to be a decreased risk of AIHA in those who received donations from a related source compared to those who received donations from an unrelated source (ARD −0.097; 95% CI −0.144, −0.051; *p* < 0.001). The included studies were found to have a low‐moderate degree of heterogeneity; however, this result was not found to be statistically significant (*I* ^2 ^= 36.17%, *p* = 0.107).

## 4 DISCUSSION

Recent studies into the risk factors associated with the development of AIHA after HSCT have had conflicting results regarding the impact of HLA matching and donor relatedness [2, 3, 4, 5, 6, 7, 8, 18, 19]. Through conducting a meta‐analysis and systematic review, however, two main findings have been established. First, patients who received HLA‐mismatched transplants were found to be more likely to develop AIHA posttransplant (ARD −0.082; 95% CI −0.157, −0.007; *p* = 0.031). Second, those who received their transplant from an unrelated donor source were also more likely to experience AIHA (ARD −0.097; 95% CI −0.144, −0.051; *p* < 0.001).

While the pathophysiology of AIHA as a complication of HSCT remains uncertain, the confirmation that HLA‐mismatched transplants lead to a higher risk of AIHA supports the suggestion that the disease may be caused by a mismatch of antigens between the donor and recipient [5]. Furthermore, Wang et al. [5] found through chimera analysis that AIHA occurs at a time when more than 95% of the patients’ cells are of donor origin, which supports speculation that aberrant immune reconstitution of donor cells within the foreign environment may favour expansion of populations of autoreactive B lymphocytes. Specifically, it is thought that perhaps an early recovery of B cells without the corresponding recovery of T regulatory cells may allow an escape of autoreactive B cells, which would otherwise be prevented through central and peripheral tolerance [4]. However, it is uncertain how donor haematopoietic stem cells from an unrelated donor source further promote this autoimmunity independent of HLA mismatching. A fuller understanding of the effect of relatedness and HLA matching would be gained through further research into the effect of haploidentical transplants on AIHA risk. In the present study, only two studies included sufficient data on haploidentical transplant, and both found it to be an independent risk factor for AIHA [3, 7]. In addition, a third study also reported haploidentical donor HSCT to be a risk factor; however, due to a failure to report overall exposure numbers to the various donor sources, this study could not be included in the meta‐analysis in this paper [4]. Therefore, further research reporting the proportion of haploidentical HSCT recipients who develop AIHA as a complication would enable a more thorough investigation of the impact of donor relatedness and HLA matching on AIHA risk. It should also be noted that most studies did not provide data on the number of HLA mismatches, which were involved in the transplants that were classified as mismatched. Further information regarding the number of mismatches could have allowed for a more detailed analysis of the risk of additional mismatches on the risk of AIHA.

Other studies have also investigated other risk factors associated with AIHA post‐HSCT and found that paediatric patients are more likely to develop AIHA posttransplant [4, 20]. These observations have shed further light on the pathophysiology and supported suggestions that insufficient T regulatory cell development in the developing thymus may lead to this autoimmune phenomenon [4]. Furthermore, paediatric patients are more likely to be receiving transplants for non‐malignant haematological disorders or metabolic disorders, which have been found to have an increased incidence of AIHA after transplant [8]. This phenomenon is perhaps because recipient have a more intact immune system than those who have previously received chemotherapy for malignancy [8]. In particular, patients with thalassaemia have been found to have a far greater risk of AIHA posttransplant, which may reflect a prior sensitisation of the immune system due to chronic blood transfusion [18].

There have also been suggestions that AIHA that develops early after HSCT is more likely to be of an IgM cold reacting class, while those that develop later are more likely to be of the IgG warm reacting class [21]. While some studies have supported this finding [4, 5, 7], others have failed to see this phenomenon [6]. Pathophysiological, it is thought that this reflects the development of immune reconstitution of the B cells in the patient posttransplant [5].

In the present study, the heterogeneity was found to be moderate in the meta‐analysis of donor relatedness and associated AIHA risk and high in the meta‐analysis of HLA matching and associated AIHA risk. The heterogeneity seen may be due to the differing populations seen in the various studies. Three of the studies included featured only adults undergoing transplant for haematological disorders, mostly malignancies, whereas the others included paediatric patients with a greater proportion of non‐malignant diseases. Furthermore, studies varied on the proportion of the various stem cell sources used, with some studies using a higher proportion of cord blood stem cells, which have previously been linked to a higher rate of AIHA [5]. Furthermore, the study by Yang et al. [7] found a significantly higher rate of AIHA in their population of HLA‐mismatched patients than other studies; however, it should be noted that this study also included a higher proportion of haploidentical transplants than other studies, which could have increased the risk substantially. It should also be noted that most studies did not provide data on the number of HLA mismatches that were involved in the transplants that were classified as mismatched. Further information regarding the number of mismatches could have allowed for a more detailed analysis of the risk of additional mismatches on the risk of AIHA.

In addition, the included studies used various definitions of AIHA, with one relying on a positive DAT and symptomatic indications of haemolysis [18], whereas others possessed more stringent definitions that included biochemical indications of haemolysis, eluate testing and DAT in order to diagnose a case [3, 6, 19]. Sensitivity to cases was also a source of variation in the studies, as some studies used routine DAT in the follow‐up [6], whereas others only tested for DAT in the event of a clinical suspicion of AIHA [8]. In order to account for the level of heterogeneity across the studies, a random effects model was used in this analysis; therefore, the impact on the results obtained is likely to be minimal.

A major limitation of this study is the lack of prospective studies on the risk of AIHA in HSCT patients. The fact that only retrospective studies were included reflects the available literature; however, it may serve to introduce bias to the study. Most of the studies included were conducted through medical record reviews of HSCT patients at various institutions; therefore, the validity of the results relies upon the accuracy of the transcription and availability of full patient records. Furthermore, some studies were conducted at referred treatment centres where patients were only seen for a year before returning to their original treatment centre; therefore, if AIHA had been diagnosed later than this, the hospital that undertook the transplant may not have been aware of these more long‐term cases [8]. Last, due to the relatively low incidence of this complication in an already small population, there is the inherent difficulty of determining prognostic factors with a low number of events [6].

The results of this study provide evidence for the need for healthcare practitioners to monitor patients receiving HSCT, especially from HLA‐mismatched and unrelated donor sources, for signs of AIHA, such as haemolysis and anaemia. Furthermore, it also suggests the superiority of HLA‐matched and related donor stem cell sources for mitigating the risk of AIHA posttransplant, which has been identified as a source of substantial morbidity and mortality in those who develop this complication [5, 7]. Additionally, this research provides further support for researchers regarding the basis of mismatched antigens being involved in the pathogenesis of AIHA post‐HSCT. As summarised in Table 4, future research should aim to further elucidate the prognostic factors associated with posttransplant AIHA by conducting large prospective studies, which have less risk of substantial bias. Studies on immune reconstitution after transplant, such as the recovery of B and T‐cell populations, could also further help to identify differences in those who go on to experience this complication and those who do not. In addition, further study is needed regarding the level of risk associated with haploidentical HSCT, which few present studies have examined.

**TABLE 4:** Recommendations for future research directions into autoimmune haemolytic anaemia (AIHA) in haematopoietic stem cell transplant (HSCT) recipients

Gap in research	Trial design	Research question
Analysis of number of HLA mismatches in HSCT recipients and the associated risk of AIHA	Prospective cohort study	Does additional HLA mismatches increase the associated risk of AIHA in HSCT recipients?
Comparison of immune reconstitution in those who experience AIHA and those who do not	Prospective cohort study	Does aberrant immune reconstitution occur in those who go on to develop AIHA? (e.g., B and T regulatory cell recovery)
Risk of AIHA in those who receive haploidentical transplants	Prospective cohort study	Does haploidentical transplants result in a higher risk of AIHA than other HLA‐mismatched sibling transplants?

## 5 CONCLUSION

Through conducting this study, it can be concluded that patients receiving HSCT from HLA‐mismatched and unrelated donor sources have a greater risk of developing AIHA. As AIHA represents a source of morbidity and mortality for patients, it is vital that healthcare practitioners be aware of the benefits of receiving an HLA‐matched HSCT from a related donor source, if this represents a possibility for the patient. Furthermore, these results also provide further confirmation of these prognostic factors for researchers hoping to elucidate the pathophysiology of AIHA post‐HSCT. However, further research is needed to investigate the effects of haploidentical transplants on AIHA risk.

## CONFLICT OF INTEREST

The authors declare they have no conflicts of interest.

## FUNDING INFORMATION

The authors received no specific funding for this work.

## ETHICS STATEMENT

We have no ethical issues to address in this systematic review and meta‐analysis.

## Data Availability

As stated we would make data availability for this review.
